# The Sustainable Bioactive Dyeing of Textiles: A Novel Strategy Using Bacterial Pigments, Natural Antibacterial Ingredients, and Deep Eutectic Solvents

**DOI:** 10.3390/gels9100800

**Published:** 2023-10-05

**Authors:** Cláudia Mouro, Ana P. Gomes, Rita V. Costa, Farzaneh Moghtader, Isabel C. Gouveia

**Affiliations:** FibEnTech Research Unit, Faculty of Engineering, University of Beira Interior, 6200-001 Covilhã, Portugal; claudia-mouro@hotmail.com (C.M.); anaritacosta23@hotmail.com (R.V.C.);

**Keywords:** bacterial pigments, crude gel prodigiosin pigment, natural dyeing, bio-mordant, L-Cysteine, gel-based deep eutectic solvent

## Abstract

The textile industry stands as a prominent contributor to global environmental pollution, primarily attributable to its extensive reliance on synthetic dyes, hazardous components, and solvents throughout the textile dyeing and treatment processes. Consequently, the pursuit of sustainable textile solutions becomes imperative, aimed at replacing these environmentally unfriendly constituents with biobased and bioactive pigments, antibacterial agents, and, notably, natural solvents. Achieving this goal is a formidable yet indispensable challenge. In this study, the dyeing ability of the crude gel prodigiosin, produced by non-pathogenic bacteria *Serratia plymuthica*, was investigated on various multifiber fabrics at different conditions (temperature and pH) and by using salts and alternative mordants (the conventional Ferrous Sulphate (FeSO_4_) and a new bio-mordant, L-Cysteine (L-Cys)). Additionally, a novel gel-based Choline chloride (ChCl)/Lactic acid (LA) (1:2) deep eutectic solvent (DES) dyeing medium was studied to replace the organic solvents. Nylon fabrics dyed with 3.0% over the weight of the fiber (*owf*) L-Cys at pH = 8.3 had improved color fastness to washing, while the gel-based ChCl/LA (1:2) DES dyebath provided a better color fastness to light. Moreover, nylon fabrics under these conditions exhibited remarkable antimicrobial activity against *Staphylococcus aureus* (*S. aureus*) and *Pseudomonas aeruginosa* (*P. aeruginosa*). In conclusion, the utilization of the crude gel-based prodigiosin pigment demonstrates a distinct advantage in dyeing textile materials, aligning with the growing consumer demand for more eco-friendly and sustainable products. Additionally, the application of the natural reducing agent L-Cys, previously untested as a bio-mordant, in conjunction with the use of gel-based DES as a dyeing medium, has showcased improved colorimetric and antibacterial properties when applied to nylon that is dyed with the crude gel prodigiosin pigment.

## 1. Introduction

In today’s context, the textile industry has emerged as a significant contributor to global pollution, primarily stemming from the utilization of synthetic and toxic dyes, as well as the various hazardous chemicals and ingredients that are employed in the dyeing and finishing processes. These practices have resulted in substantial adverse environmental consequences [1,2]. Notably, textile dyeing procedures discharge pollutants into water, soil, and the atmosphere, while also demanding excessive amounts of water and energy, thereby exerting a profoundly detrimental impact on both the environment and human health [1,2,3,4].

In light of these circumstances, and in response to increasingly stringent government policies and regulations, there arises a critical need to develop and implement strategies that are more environmentally friendly and economically sustainable [2,5]. Furthermore, consumers are displaying a heightened concern for environmental issues within the textile industry, demonstrating a willingness to invest in materials that are less harmful and less environmentally toxic [5].

Thus, the re-introduction of natural dyes, as an alternative option to the petrochemical-based synthetic dyes that have been dominating the modern textile industry since their arise due to their low manufacturing costs, processability, and desirable coloring characteristics, has been explored to achieve economically and environmentally sustainable dyeing processes [1,2,3]. In fact, the recent interest in natural dyes over dangerous synthetic dyes is due to their better biodegradability, non-toxicity, environmental safety, and lower incidence of allergic reactions [1,3,4,5]. Nevertheless, natural dyes, particularly those derived from plants, display a few inherent drawbacks, such as a low affinity for the textiles, poor color fastness properties, a limited range of color, low yields, problems in the reproduction of shades, long extraction processes, and seasonal variations [1,3]. Accordingly, to overcome such limitations and improve the textile dyeing processes with green natural dyes, different mordants, such as metallic salts and natural tannin-containing plants, have been applied to enhance the dyeability and fastness features of the textile materials [1,3]. However, most of these mordants usually alter the hue and darkness or brightness of the dyed textile fibers [1]. Therefore, in this study, the amino acid L-Cysteine (L-Cys), widely used as a reducing agent, was tested as a new natural bio-mordant, which has not been applied before to the best of our knowledge. Additionally, gel-based deep eutectic solvents (DESs), formed by a hydrogen bond donor and a hydrogen bond acceptor, which have been aimed to be used as novel environmentally friendly and water-free dyeing mediums, gained our attention due to their ability to improve color fastness properties without changing dyeing properties [6,7].

In turn, the high demand for natural dyes has led to the analysis of new sources for their extraction [2,3,5]. Among them, microbial pigments have been highlighted by several authors due to their diversity, greater productivity, rapid growth in a culture medium, and efficient control of the fermentation process [3]. Moreover, it is worth noting that microbial pigments can be cultivated using cost-effective fermentation substrates, specifically agro-industrial residues [8,9,10].

Microbial pigments can be produced by bacteria, fungi, yeast, and algae, with various chemical structures and a large array of colors [3]. Moreover, the pigments that are produced from natural sources present interesting bioactive properties in addition to their color features, such as antioxidant, antimicrobial, and anticancer activity, as well as being insect-repellent and providing ultraviolet protection [1,2,3,4,11,12].

Over the years, a wide range of bacteria have been reported for their ability to synthesize multifunctional secondary metabolites in response to external stimuli, with a high growth rate [2,11,12]. Concerning that, this study aimed to dye a multifiber fabric with a crude gel prodigiosin pigment, a bacterial metabolite that is commonly produced by numerous species, including *Serratia* sp., and which is recognized for its attractive red color and a broad spectrum of bioactive properties, such as antimicrobial, antifungal, anticancer, antimalarial, and immunosuppressive activities [2,3,12]. Furthermore, the prodigiosin pigment has been widely extracted and purified using conventional organic solvents, like acidified ethanol and methanol [13,14,15,16,17,18,19,20,21], chloroform [17,21], acetonitrile [17,21], dimethyl sulfoxide (DMSO) [14,17,21], ethyl acetate [18], petroleum ether [18], diethyl ether [18], and acetone [14,18], which are harmful to human health and the environment. Accordingly, to the authors’ knowledge, the present study is the first report to use a crude gel prodigiosin pigment obtained directly from medium-agar plates, without further adopting solvent extraction and purification methods. Moreover, the effect of the dyeing conditions, such as the temperature, the salts (sodium hydroxide (NaOH) and sodium hydrosulphite (Na_2_S_2_O_4_)), and mordant (Ferrous Sulphate (FeSO_4_) and L-Cys) addition, as well as the use of a gel-based Choline chloride (ChCl)/Lactic acid (LA) (1:2) DES as a dyeing medium, were investigated. Furthermore, the crude gel prodigiosin pigment, collected directly from the culture medium, was revealed to have several economic and environmental benefits, and a high potential to replace the synthetic dyes that are used in the textile industry. This is because the prodigiosin pigment exhibits good biodegradability and low toxicity, in comparison with synthetic dyes derived from non-renewable sources or petrochemicals, which are harmful and toxic to the human body and the environment [3]. Moreover, prodigiosin pigment production via fermentation is relatively inexpensive and may use natural substrates, such as agro-industrial waste, which are cost-effective and sustainable [22]. Furthermore, previous studies have reported that prodigiosin exhibits very good fastness properties and a brilliant color in dyed textiles, highlighting the possibility of bacterial pigments as natural source of dyes for the textile industry to replace synthetic dyes [2,3,23].

Furthermore, the novel bio-mordant, L-Cys, exhibited a favorable green dyeing performance, particularly on nylon, and demonstrated its capability to enhance the inherent antibacterial characteristics of the prodigiosin pigment. Additionally, the utilization of a water-free gel-based deep eutectic solvent ChCl/LA (1:2) as a dyeing medium effectively enhanced the lightfastness properties of dyed nylon fabrics, further bolstering their outstanding antibacterial activity. Therefore, the sustainable dyeing process for dyeing nylon with the crude gel prodigiosin pigment using a DES can significantly reduce water consumption and effluent emission, which is crucial to develop novel green dyeing solutions.

## 2. Results and Discussion

### 2.1. Evaluation of the Color Strength and Equalization of the Dyed Fabrics

#### 2.1.1. The Effect of Temperature

**Figure 1:**
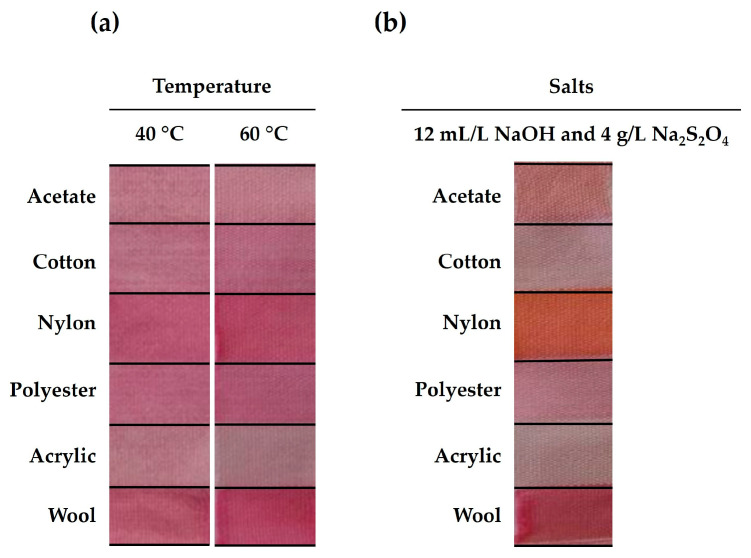
Influence of temperature (**a**) and of the addition of salts (sodium hydroxide (NaOH) and sodium hydrosulphite (Na_2_S_2_O_4_)) (**b**) on multifiber dyeing with the crude gel prodigiosin pigment.

The multifiber fabrics that were dyed with the crude gel prodigiosin pigment at 40 °C and 60 °C revealed various tones for the different textile fibers (Figure 1a). Such occurrences were essentially due to the different interactions that were established between the pigment and the fiber, as well as the color of the fiber itself. In addition, the performance of the bacterial pigments, namely the color depth and dyeing rate, depends significantly on the dyebath temperature [2].

According to the literature, high temperatures above 80 °C are more suitable for dyeing textile fibers with prodigiosin [24,25,26,27]. However, more environmentally friendly and sustainable dyeing methods have emerged as a more efficient and economical alternative to conventional processes. Concerning that, lower temperatures, like 40 °C and 60 °C, were applied, and their effect on the color strength (*K/S*) of the dyed multifiber fabrics was evaluated, and is and summarized in Table 1a. The temperatures of 40 °C and 60 °C showed similar results for the multifiber fabrics. Nevertheless, the crude gel prodigiosin pigment presented a greater affinity for the wool and nylon fibers (*K/S_Wool_40 °C_* = 1.56; *K/S_Wool_60 °C_* = 1.38; *K/S_Nylon_40 °C_* = 1.17; *K/S_Nylon_60 °C_* = 1.20). Plus, more uniform colors were observed at 60 °C, resulting in an improvement in the dyeing equalization (*dE*) values (*dE_Wool_60 °C_* = 0.05; *dE_Nylon_60 °C_* = 0.04). Therefore, 60 °C was chosen to achieve a better crude gel prodigiosin dyeing effectiveness.

#### 2.1.2. The Effect of the Dyeing Aids Addition

##### The Addition of Salts

In order to improve the crude gel prodigiosin pigment’s solubility, NaOH and Na_2_S_2_O_4_ were used as an alkali and a reducing agent, respectively. Regarding that, the addition of these aides changed the dye bath’s pH from 7.0 to 12.5, resulting in different interactions between the crude gel prodigiosin pigment and several fibers (Figure 1b). The nylon fabric presented the most significant difference, exhibiting a fluorescent orange tone. This essentially occurred because the prodigiosin pigment is sensitive to pH changes, which results in color changes to a blue–purple tone at acidic pHs, pink–red at neutral pHs, and an orange–yellow tone at alkaline pHs [25,27,28]. Nevertheless, the crude gel prodigiosin pigment showed a greater affinity for nylon (*K/S_Nylon_* = 1.25) and wool (*K/S_Wool_* = 1.60) fibers (Table 1b). However, the *dE* of the different fabrics (Table 1b) was higher than that obtained for the dyed samples without auxiliaries at 60 °C (Table 1a), resulting in less homogeneous dyeing samples. Hence, the addition of these salts did not prove to be beneficial for the dyeing process with the crude gel prodigiosin pigment.

##### The Addition of Mordants

**Figure 2:**
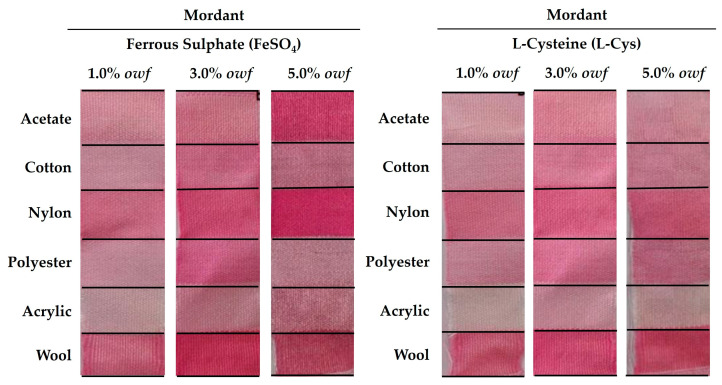
Influence of the addition of mordants (Ferrous Sulphate (FeSO_4_) and L-Cysteine (L-Cys)) at 1.0, 3.0, and 5.0% over the weight of the fiber (*owf*) on multifiber dyeing with the crude gel prodigiosin pigment.

Mordants are often used as additives in dyebaths to improve the binding of natural pigments to fibers. Concerning that, mordants form complexes with natural dyes that are capable of attaching to the fiber surfaces, improving the exhaustion of dyebaths and the washing fastness of the dyed fabrics. Nevertheless, the various forms and types of mordants can influence the shades and hues of the fabrics, resulting in brighter or darker colors, or even differences in the natural color of the pigment, which can be a beneficial or an unwanted effect [2,3]. Moreover, the mordant concentration is also a crucial factor that can cause an increase in the color intensity during textile dyeing [3]. Therefore, the effect of the addition of mordants, like FeSO_4_ and L-Cys, on the *K/S* values and *dE* of the dyed fabrics were investigated at three different concentrations (1.0%, 3.0%, and 5.0% over the weight of the fiber (*owf*)) (Figure 2 and Table 2).

To achieve bright and strong colors with natural dyes, metallic mordants such as the conventional FeSO_4_, which is known as a blue–green vitriol water-soluble dulling mordant, have been commonly used [3]. However, the solubility of the FeSO_4_ mordant in the aqueous crude gel prodigiosin pigment bath was low, resulting in a heterogeneous coloration at a rate that was directly proportional to the increase in the mordant concentration (Figure 2). Similar results were previously reported by Burkinshaw et al. and Mongkholrattanasit et al., who observed a shade of dark greyish-brown in a fabric of relatively low *K/S* when a FeSO_4_ mordant was added [29,30]. This essentially occurred due to the ability of the pigment molecules to form a metallic complex with positively charged ions (Fe^2+^), as previously described by Uddin [31]. Hence, when the FeSO_4_ mordant was added to the crude gel prodigiosin dyebath solution, different shades of color were observed in the distinct fibers, and a blackening of the color shades was revealed when the mordant concentration increased from 1.0% *owf* to 5.0% *owf* (Figure 2). Similar observations have also been reported by other researchers in the literature [3]. Moreover, a lower concentration of FeSO_4_ (1.0% *owf*) resulted in a smoother and similar dyeing among the fabrics, while a higher concentration of FeSO_4_ (5.0% *owf*) presented a deep but uneven dyeing (Figure 2 and Table 2). 

In addition, the crude gel prodigiosin pigment with 1.0% and 3.0% *owf* of FeSO_4_ exhibited the highest affinity for nylon and wool fibers (Table 2), resulting in the highest *K/S* values (*K/S_Nylon_1.0% FeSO4_* = 1.39; *K/S_Nylon_3.0% FeSO4_* = 2.02; *K/S_Wool_1.0% FeSO4_* = 1.47; *K/S_Wool_3.0% FeSO4_* = 2.44), as well as lower *dE* values (*dE_Nylon_1.0% FeSO4_* = 0.09; *dE_Nylon_3.0% FeSO4_* = 0.09; *dE_Wool_1.0% FeSO4_* = 0.10; *dE_Wool_3.0% FeSO4_* = 0.11).

On the other hand, bio-mordants like L-Cys have been regarded as a sustainable and ecological alternative to metallic mordanting. The dyeing of fabrics with the crude gel prodigiosin pigment using L-Cys as bio-mordant resulted in different tones of pink. These different tones were similar for the three different concentrations of prodigiosin (Figure 2). Nevertheless, nylon and wool displayed a better color strength (*K/S_Nylon_1.0% L-Cys_* = 1.12; *K/S_Nylon_3.0% L-Cys_* = 1.72; *K/S_Nylon_5.0% L-Cys_* = 1.61; *K/S_Wool_1.0% L-Cys_* = 1.69; *K/S_Wool_3.0% L-Cys_* = 2.08; *K/S_Wool_5.0% L-Cys_* = 2.48), and an improved dyeing equalization (*dE*) in the presence of 3.0% *owf* of L-Cys (*dE_Nylon_3.0% L-Cys_* = 0.02 and *dE_Wool_3.0% L-Cys_* = 0.04).

Therefore, as efficient dyeing depends on the color intensity of the dyed fibers and a good equalization, 3.0% *owf* of L-Cys should be chosen to achieve a better dyeing process with the crude gel prodigiosin pigment.

**Figure 3:**
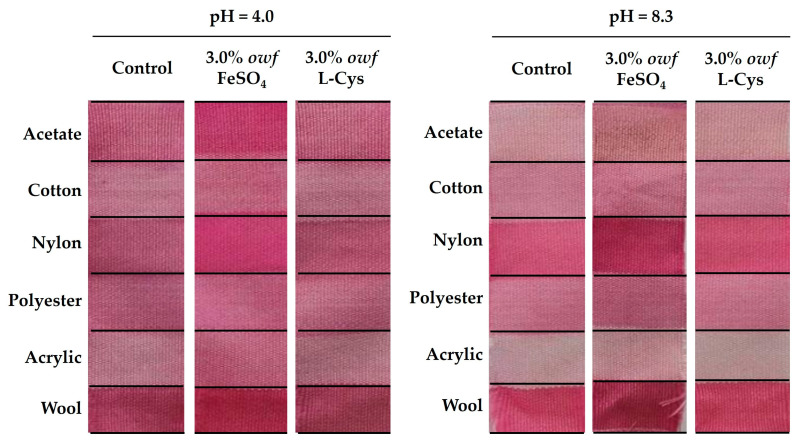
Multifiber dyeing with and without the addition of mordants (3.0% *owf* FeSO_4_ and 3.0% *owf* L-Cys) at pH = 4.0 and pH = 8.3.

Nevertheless, bacterial pigments such as prodigiosin are sensitive to pH alterations, resulting in color changes. Namely, pink prodigiosin pigment at an acidic pH changes to blue–purple, and at alkaline pHs it changes to orange–yellow [25,27]. Thus, in order to analyze the effect of the dyebath pH on the color characteristics, two different pH values (pH = 4.0 and pH = 8.3) were studied for the various types of fiber under different conditions (without and with the simultaneous mordanting of 3.0% *owf* of FeSO_4_ and L-Cys) (Figure 3 and Table 3).

At pH = 4.0, the crude gel prodigiosin pigment dyeing of the fabrics with the metallic mordant FeSO_4_ was favored in comparison with the dyeing process with bio-mordant L-Cys and without the addition of mordant, revealing a greater pink color strength, i.e., higher *K/S*, less fiber oxidation, and more efficient dyeing (Figure 3). Moreover, dyeing with FeSO_4_ demonstrated a superior stability of the pink color of crude gel prodigiosin pigment in the different fibers, particularly in nylon and wool (*K/S_Nylon_3.0% FeSO4_pH = 4.0_* = 3.30; *K/S_Wool_3.0% FeSO4_pH = 4.0_* = 3.22; *dE_Nylon_3.0% FeSO4_pH = 4.0_* = 0.13; *dE_Wool_3.0% FeSO4_pH = 4.0_* = 0.35). Therefore, the acidic pH provided more efficient dyeing in the presence of the metallic mordant, increasing its solubility and improving its equalization (Table 3). Similar data were reported by Uddin, who revealed that mordants’ metal ions act as electron acceptors for electron donors and produce coordination bonds with the dye molecules, making them insoluble in water [31]. In this way, metallic mordants, such as FeSO_4_, can improve the dye uptake and retention, resulting in a higher depth of shade and greater color fastness properties. Furthermore, Alihosseini et al. showed that prodigiosin pigments produced from a strain of Vibrio sp. that was isolated from marine sediments were able to dye wool, nylon, acrylics, and silk fibers [24]. However, Alihosseini et al. found enhanced dyeing characteristics. The reason for this was that different prodigiosin-producing strains were used, and the prodigiosin pigments exhibited different levels of purification [24]. Thus, the bacterial pigment color can be influenced by their nature and, consequently, various shades of the same pigment can be obtained on similar fabrics [2].

On the other hand, the infeasibility of dyeing with the crude gel prodigiosin pigment at an acidic pH for the fibers that were dyed simultaneously with L-Cys and without the addition of mordant was confirmed with the high values of *dE* and, consequently, resulted in a lower equalization of dyeing.

In turn, at pH = 8.3, the dyeing process with the crude gel prodigiosin pigment was favored in the three different conditions (without the addition of mordants, with the addition of L-Cys, and with FeSO_4_) as shown in Table 3. However, the fibers dyed with the crude gel prodigiosin pigment in the presence of L-Cys and without the addition of any auxiliary revealed similar behavior (Figure 3 and Table 3). Nonetheless, the dye was more homogeneous when L-Cys was added, revealing good equalization values (*dE_Acetate_3.0% L-Cys_pH = 8.3_* = 0.11; *dE_Cotton_3.0% L-Cys_pH = 8.3_* = 0.13; *dE_Nylon_3.0% L-Cys_pH = 8.3_* = 0.05; *dE_Polyester_3.0% L-Cys_pH = 8.3_* = 0.11; *dE_Acrylic_3.0% L-Cys_pH = 8.3_* = 0.11; *dE_Wool_3.0% L-Cys_pH = 8.3_* = 0.27), due to the association of the pH with the pKa of L-Cys.

On the other hand, when FeSO_4_ was used, the oxidation of the fibers increased, mainly in the wool and nylon, due to the higher adsorption to the surface of the fibers. Nevertheless, although a stronger color strength for the nylon and wool fibers was obtained (*K/S_Nylon_3.0% FeSO4_pH = 8.3_* = 3.67; *K/S_Wool_3.0% FeSO4_pH = 8.3_* = 3.11), the *dE* was higher (*dE_Nylon_3.0% FeSO4_pH = 8.3_* = 0.11; *dE_Wool_3.0% FeSO4_pH = 8.3_* = 0.33) (Figure 3 and Table 3).

Hence, the choice of the ideal conditions for the dyeing process with the crude gel prodigiosin pigment depends directly on the uniformity of the dyes, since the color intensity may not result in a better dyeing performance, with this being difficult to determine qualitatively through observations of the dyed fabrics. So, it was decided to proceed with 3.0% *owf* L-Cys at pH = 8.3 for dyeing the nylon and wool fabrics, which exhibited higher *K/S* values and lower *dE* values.

#### 2.1.3. The Effect of the Use of Gel-Based Deep Eutectic Solvents (DESs) as a Prodigiosin Dyeing Medium

**Figure 4:**
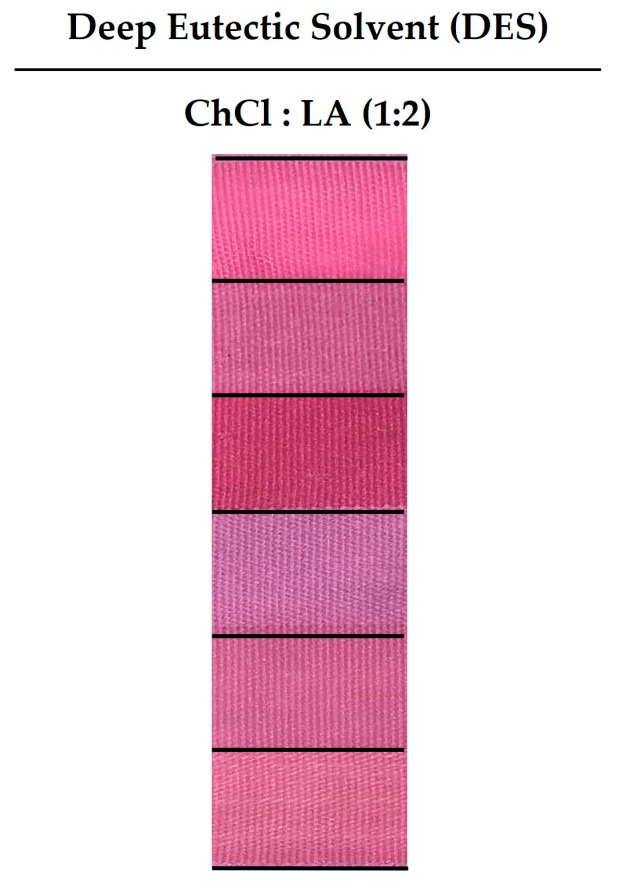
Influence of the use of a gel-based deep eutectic solvent (DES) as a dyeing medium on the dyeing of multifiber fabric with the crude gel prodigiosin pigment.

Recently, gel-based DESs have received increasing attention as a promising green alternative to conventional solvents in order to extract specific alkaloid compounds from different sources, namely from plants, like proline/oxalic acid (1:1), ChCl/LA (1:2), and ChCl/Fructose (5:1) [32,33,34]. In addition, gel-based DESs that are prepared from a hydrogen bond acceptor and a hydrogen bond donor, such as ChCl/urea/glycerol and ChCl/ethylene glycol, can act as water-free solvents that are able to dissolve dyes without changing their dyeing properties [6]. Hence, in this study, the gel-based ChCl/LA (1:2) DES mixture was selected as a dyeing medium for the first time to dissolve the crude gel prodigiosin pigment, a bacterial alkaloid, as it has previously been used to extract alkaloids from plants. According to Figure 4 and Table 4, the multifiber fabric dyed with the crude gel prodigiosin pigment, dissolved using the gel-based ChCl/LA (1:2) DES with pH = 1.7, exhibited different shades. Additionally, the strongest red–pink color was obtained in nylon (*K/S_Nylon_gel-based ChCl/LA (1:2) DES_* = 3.33). This value was higher that those found for the nylon dyed with the crude gel prodigiosin pigment containing 3.0% *owf* L-Cys at pH = 8.3 (*K/S_Nylon_3.0% L-Cys_pH = 8.3_* = 2.30). Moreover, the ChCl/LA (1:2) DES-based gel, used to dissolve the crude gel prodigiosin pigment, increased the final equalization in the dyeing process of the multifiber fabric, reaching lower *dE* values for the different fibers (*dE_Acetate_gel-based ChCl/LA (1:2) DES_* = 0.02; *dE_Cotton_gel-based ChCl/LA (1:2) DES_* = 0.02; *dE_Nylon_gel-based ChCl/LA (1:2) DES_* = 0.01; *dE_Polyester_gel-based ChCl/LA (1:2) DES_* = 0.01; *dE_Acrylic_gel-based ChCl/LA (1:2) DES_* = 0.01; *dE_Wool_gel-based ChCl/LA (1:2) DES_* = 0.01), in comparison with the values obtained for the fabrics that were dyed with the crude gel prodigiosin pigment containing 3.0% *owf* of L-Cys at pH = 8.3 (*dE_Acetate_3.0% L-Cys_pH = 8.3_* = 0.11; *dE_Cotton_3.0% L-Cys_pH = 8.3_* = 0.13; *dE_Nylon_3.0% L-Cys_pH = 8.3_* = 0.05; *dE_Polyester_3.0% L-Cys_pH = 8.3_* = 0.11; *dE_Acrylic_3.0% L-Cys_pH = 8.3_* = 0.11; *dE_Wool_3.0% L-Cys_pH = 8.3_* = 0.27).

Therefore, these results demonstrate the potential of the gel-based DES to act as a water-free dyeing medium that is able to decrease the enormous amounts of water required for the dyeing process. In addition, these new dyeing mediums can simultaneously be used as a mordant, providing better absorption and fastness properties [3,6]. For example, Zheng et al. used a ChCl/Ethylene glycol DES to dissolve disperse dyes without changing their chemical structure and original dyeing properties. Moreover, they revealed that after dissolving the dyes into the DES mixture, the particle sizes of the dispersed dyes were significantly decreased, improving their permeability and, consequently, the *K/S* values of the polyester fabrics, as well as the wet rubbing fastness [6]. In fact, their findings showed that green dyeing processes arise as a highly promising approach to reducing water pollution and dyeing costs, as well as meeting the demand for environmental protection.

### 2.2. Analysis of the Surface Morphology of Dyed Fabrics through SEM

**Figure 5:**
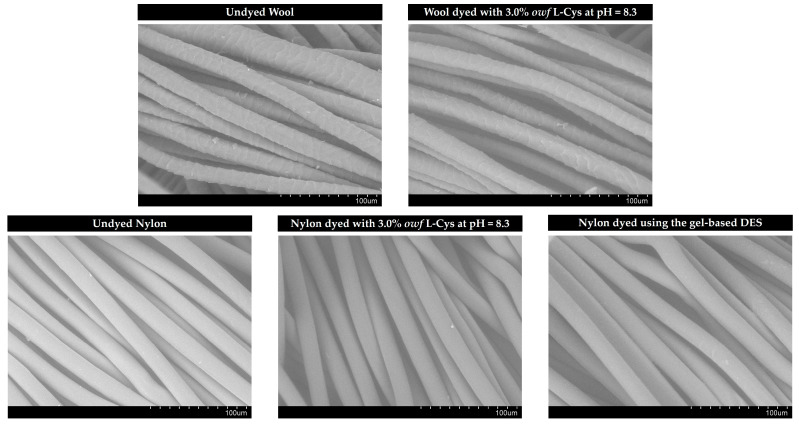
Characterization of the undyed and dyed fabrics’ surface morphological properties. SEM images of the wool before and after dyeing with 3.0% *owf* L-Cys at pH = 8.3, as well as the nylon before and after dyeing with 3.0% *owf* L-Cys at pH = 8.3 and using the gel-based ChCl/LA (1:2) DES.

SEM analysis was performed to analyze the surface morphology of both the undyed and dyed fabrics with superior performance, namely the wool and nylon fabrics that were dyed with 3.0% *owf* L-Cys at pH = 8.3, and the nylon dyed using the gel-based ChCl/LA (1:2) DES as a dyeing medium. Figure 5 shows that the wool fabrics, both undyed and dyed with the crude gel prodigiosin pigment in different conditions, displayed a smooth morphology. Similar observations were also recorded after the analysis of the surface of the nylon fabrics. Therefore, the SEM images evidenced that no changes were observed between the undyed and dyed fabrics with the crude gel prodigiosin pigment (Figure 5).

### 2.3. Evaluation of Fastness Properties of Dyed Fabrics

#### 2.3.1. Assessment of the Washing Fastness Properties

In this section, the washing fastness of the fabrics that obtained the deepest coloration and better uniformity was evaluated, i.e., the nylon and wool dyed with the crude gel prodigiosin pigment with 3.0% *owf* of L-Cys at pH = 8.3, and the nylon dyed using the gel-based ChCl/LA (1:2) DES as a dyeing medium. These fabrics were evaluated before and after washing, according to ISO 105-C06:2010, and the color difference (∆E*) and color fastness (*∆Color*) were determined (Table 5).

The obtained results showed that the selected fabrics displayed a relatively high ∆E*, i.e., higher than 1, which reveals that a significant color difference was observed after washing, particularly when the nylon was dyed with the crude gel prodigiosin pigment using the gel-based ChCl/LA (1:2) DES as a dyeing medium (∆E*_Nylon_gel-based ChCl/LA (1:2) DES_ = 11.12). In addition, the ∆L* parameter, which measures the lightness difference, was also considered to be an important factor, since the lightness (*L*) provides a suitable roadmap for the depth of shade, particularly for the colors that easily change shade and consequently exhibit high values of ∆a* and ∆b* after washing [25]. Concerning this, it is possible to identify a satisfactory washing fastness for the nylon and wool that were dyed with the crude gel prodigiosin pigment with 3.0% *owf* of L-Cys bio-mordant at pH = 8.3 (∆L*_Nylon_3.0% L-Cys_pH = 8.3_ = 1.66 and ∆L*_Wool_3.0% L-Cys_pH = 8.3_ = 3.31). However, the nylon dyed with the gel-based ChCl/LA (1:2) DES dyebath presented poor washing fastness (∆L*_Nylon_gel-based ChCl/LA (1:2) DES_ = 11.12), which was confirmed through a visual assessment of the fabrics, where the washed samples were set side by side with the unwashed ones in the Color-Chex lightbox, and the *∆Color* was evaluated according to the CIELab gray-scale values, using the CIE illuminant D65. In fact, a better fastness index was observed for the nylon dyed with 3.0% *owf* of L-Cys at pH = 8.3 (*∆Color* = 3) in comparison to the wool, which revealed a lower fastness index (*∆Color* = 2) (Table 5). Furthermore, the nylon that was dyed using the gel-based ChCl/LA (1:2) DES showed the lowest fastness index (*∆Color* = 1–2), resulting in a greater release of pigment during the washing.

Therefore, the nylon dyed with 3.0% *owf* of L-Cys at pH = 8.3 retained a higher amount of crude gel prodigiosin pigment on its surface that was not lost so much after washing, making the prodigiosin dyeing process more efficient. Such data are in accordance with the results published by Liu et al., which reported that acrylics and polyamide (nylon) dyed with a high-purity prodigiosin of the mutant strain of *Serratia marcescens* jx1-1 showed good washing fastness [26].

#### 2.3.2. Assessment of the Light Fastness Properties

**Figure 6:**
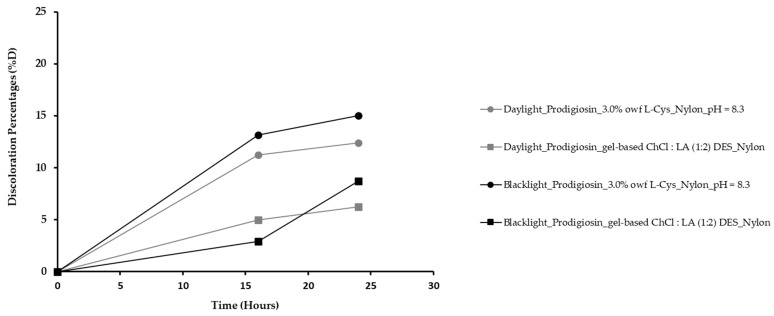
Evaluation of the light fastness properties of the nylon dyed with the crude gel prodigiosin pigment, with 3.0% *owf* of L-Cys at pH = 8.3 and using the gel-based ChCl/LA (1:2) DES. Discoloration percentages (*%D*) of each sample after daylight and blacklight exposure.

Commonly, colored fabrics after light exposure fade upon the presence of oxygen and humidity. In addition, bacterial or natural pigments exhibit poor light stability in comparison with most synthetic dyes [3]. Therefore, in this study, the nylon samples that were dyed with the crude gel prodigiosin pigment containing 3.0% *owf* of L-Cys at pH = 8.3 and using the gel-based ChCl/LA (1:2) DES were directly exposed to blacklight and daylight for 0, 16, and 24 h to accelerate their fading, as shown in Figure 6 through the discoloration percentages (*%D*).

After 24 h, the color fading was similar for both samples incorporated with the L-Cys bio-mordant due to the L-Cys antioxidant-reducing power. However, the nylon dyed with prodigiosin at pH = 8.3, exposed to artificial daylight and containing L-Cys, suffered a slightly lower color change in comparison with the blacklight sample. Thus, for the nylon containing L-Cys as bio-mordant, it is proposed that L-Cys oxidizes and forms cystine, which is more susceptible to UV light and less to other lights, resulting in lower discoloration percentages [35]. Moreover, Koch et al. revealed that L-Cys is more susceptible to dark light [36]. This result is consistent with the slight increase in the *%D* for the nylon dyed with the crude gel prodigiosin pigment containing 3.0% *owf* of L-Cys at pH = 8.3 and exposed to blacklight (Figure 6). Therefore, the addition of the L-Cys bio-mordant can improve the fastness to light of nylon fibers dyed with prodigiosin, a photosensitive pigment, and makes this work innovative to obtain better natural dyeing. Furthermore, the samples dyed with the crude gel prodigiosin pigment using the gel-based ChCl/LA (1:2) DES exhibited a very good light fastness after being exposed to blacklight and daylight, reaching *%D* of 8.70% and 6.22%, after 24 h, respectively. Furthermore, the obtained results show that the nylon dyed with the crude prodigiosin pigment using the gel-based ChCl/LA (1:2) DES presented light fastness properties similar to those found in the nylon dyed with 3.0% *owf* of L-Cys at pH = 8.3, being more susceptible to blacklight (Figure 6). However, an improvement in the color’s light fastness was observed due to the incorporation of the gel-based ChCl/LA (1:2) DES as a dyebath, thus confirming its capability to improve colorfastness to light.

### 2.4. Evaluation of the Antibacterial Activity of Dyed Fabrics

The antibacterial activity of the nylon fabrics dyed with the crude gel prodigiosin pigment, both with and without the addition of 3.0% *owf* of L-Cys at pH = 8.3, as well as using the gel-based ChCl/LA (1:2) DES, was determined against two bacteria, *Staphylococcus aureus* (*S. aureus*) (Gram-positive) and *Pseudomonas aeruginosa* (*P. aeruginosa*) (Gram-negative), after 24 h.

**Figure 7:**
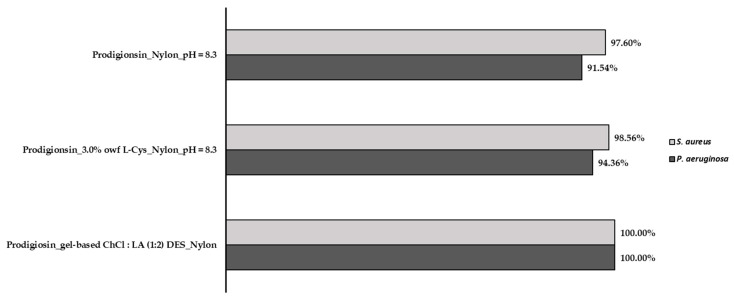
Antibacterial activity evaluation: the percentage of bacterial reduction of the nylon dyed with the crude gel prodigiosin pigment with and without the addition of 3.0% *owf* of L-Cys at pH = 8.3 and using the gel-based ChCl/LA (1:2) DES against *Staphylococcus aureus* (*S. aureus*) and *Pseudomonas aeruginosa* (*P. aeruginosa*).

According to the results presented in Figure 7, a positive inhibitory effect against both strains was shown. Both nylon samples that were dyed with the crude gel prodigiosin pigment, both with and without L-Cys, exhibited greater efficacy against Gram-positive bacteria (*S. aureus*), demonstrating an inhibitory effect of 98.56% and 97.60%, respectively. On the other hand, a lower inhibitory effect was observed for the Gram-negative bacteria (*P. aeruginosa*) (94.36% for the nylon dyed with the prodigiosin pigment containing 3.0% *owf* of L-Cys and 91.54% for the nylon dyed with the crude gel prodigiosin pigment, without the addition of auxiliaries). These results are in accordance with data that were previously reported by Ren et al. and Metwally et al., who reported that prodigiosin exhibits the ability to induce the production of autolysins in Gram-positive bacteria, like *S. aureus*, causing cell lysis and, consequently, cell death [3,37]. Plus, prodigiosin pigment can penetrate the cell membrane and inhibit specific target enzymes, like topoisomerase IV and DNA gyrase, avoiding cell growth [3]. However, this effect is not observed in Gram-negative bacteria, like *P. aeruginosa*. For this type of bacteria, prodigiosin can hinder RNA and protein synthesis, cell division, membrane integrity, and cellular respiration [3].

Moreover, the nylon dyed with the crude gel prodigiosin pigment with the addition of L-Cys displayed an enhanced antibacterial effect in comparison with the nylon without the addition of auxiliaries, like the L-Cys bio-mordant. This result can be explained by taking into account the high reactivity between the thiol groups (-SH) of L-Cys and the -SH present in the cell membrane, which leads to a drastic decrease in the enzymatic activity and bacterial metabolism, causing the leakage of cellular content and cell death [38].

In turn, the obtained results show that the nylon dyed with the crude gel prodigiosin pigment using the gel-based ChCl/LA (1:2) DES presented a superior inhibitory effect on bacterial growth, resulting in an antibacterial efficiency of 100% for *S. aureus* and *P. aeruginosa*, when compared to the control group that did not receive prodigiosin dyeing and the use of auxiliaries. The percentage of inhibition of the *S. aureus* and *P. aeruginosa* growth is in accordance with the data noticed by Mouro et al., where an inhibitory effect of 100% was observed against *S. aureus* and *K. pneumoniae* when nanofibers of polyvinyl alcohol (PVA) were blended with a L-Cys/LA DES containing dissolved wool keratin [39]. In addition, in the literature, it has been described that ChCl-based DESs improve the antibacterial activity against several Gram-positive and Gram-negative bacteria [40]. Moreover, LA has been demonstrated to be lethal to microorganisms due to its ability to diffuse to the inside of the cell through the cell membrane and ionize. Also, the acidic pH can result in DNA damage and affect the interactions with the membrane enzymes and proteins, causing changes in the cell membrane structure and permeability, and leading to the leakage of the cellular constituents and cell death [41].

## 3. Conclusions

Conventionally, textiles are dyed with synthetic dyes which are very effective but, unfortunately, cause severe environmental pollution. Bacterial pigments are important alternatives; however, they are traditionally extracted using toxic organic solvents that are hazardous to the environment. In this study, the crude gel of prodigiosin pigment, produced from non-pathogenic bacteria *Serratia plymuthica*, was successfully used to dye multifiber fabrics in order to develop an eco-friendly and efficient dyeing process, as an alternative to those hazardous substances and protocols for a sustainable future. The influence of different parameters was studied, including the effects of temperature and the addition of salts (NaOH and Na_2_S_2_O_4_), and the pH was changed by using two mordants (FeSO_4_ and L-Cys) with different compositions. Plus, the use of a gel-based ChCl/LA (1:2) DES as a promising water-free dyeing medium was studied.

The attained results show that the functionalization process at 60 °C for 60 min, using 3.0% *owf* of L-Cys at pH = 8.3, was suitable to obtain colored materials, particularly nylon and wool fabrics, with high *K/S* values and improved *dE* values, as well as nylon fibers that were dyed using the gel-based ChCl/LA (1:2) DES as a dyeing medium. In addition, the nylon dyed with 3.0% *owf* of L-Cys at pH = 8.3 displayed a superior fastness to washing, while the use of the gel-based ChCl/LA (1:2) DES demonstrated exceptional light fastness. Moreover, the nylon dyed with the crude gel prodigiosin pigment, with the addition of the bio-mordent L-Cys and using the water-free gel-based DES, exhibited remarkable antibacterial activity against *S. aureus* and *P. aeruginosa*. Furthermore, the pink/red color of the crude gel prodigiosin pigment was revealed to be stable in the studied pH values, namely at pH = 1.7, 4.0, and 8.3, with no perceptible color change, leading to uniform dyeing with low *dE* values.

Therefore, it was concluded that the crude gel prodigiosin pigment, applied without the use of solvent extraction processes for pigment recovery, was potentially useful for dyeing nylon fabrics. In addition, the use of L-Cys—proposed for the first time in the related literature—as a bio-mordant was demonstrated to be a sustainable and ecological alternative to metallic mordants according to the color features, fastness properties, and antibacterial capability. Likewise, the use of water-free dyeing media via green solvent solutions, like the gel-based ChCl/LA (1:2) DES, can help to reduce water pollution and wastewater, which means reduced environmental impacts.

It was proposed that a screening of suitable gel-based DESs, namely composed of amino acids like L-Cys, should be evaluated in the near future as water-free and green dyeing mediums in order to develop environmentally friendly dyeing processes. In addition to the large number of potential combinations that may form potential gel-based DES dyeing media, ultrasound-assisted dyeing methods should also be investigated as an opportunity to save electrical energy and thermal energy by varying the process parameters of the dyeing process in the textile industry.

However, even though prodigiosin exhibited promising results to be used on an industrial scale, to implement this approach widely in industry, it is still necessary to overcome some limitations. Namely, in the near future, it is important to improve fermentation protocols to maximize the pigment yield and minimize production costs, particularly by using agro-industrial wastes that are available in abundant amounts. Additionally, a greater regulatory and consumer acceptance of applied bacterial pigments in textiles, such as prodigiosin, should be outlined. Furthermore, although bacterial pigments are a natural source of color, it is important to carry out further studies to evaluate their stability and colorfastness properties in textiles, in order to bring sustainability to the textile industry and improve its environmental impact and safety.

## 4. Materials and Methods

### 4.1. Materials

SDC Mutifiber DW was manufactured by James Heal. Sodium chloride (NaCl), ethanol, dipotassium hydrogen phosphate (K_2_HPO_4_), DL-Lactic acid 90% (LA), and Choline chloride 99% (ChCl) were acquired from Fisher Scientific (Fisher Scientific, Leicestershire, UK). Ferrous sulphate (FeSO_4_), L-Cysteine (L-Cys), sodium hydroxide (NaOH), sodium dithionite (Na_2_S_2_O_4_), peptone, nutrient agar (NA), nutrient broth (NB), brain heart infusion agar (BHI), hydrochloric acid (HCl), glycerol, and tween-80 were purchased from Sigma-Aldrich (Sigma-Aldrich, St. Louis, MO, USA). Agar-agar was acquired from Labkem (Labkem, Barcelona, Spain). The ECE reference detergent was provided by Bayer.

### 4.2. Production and Recovery of Prodigiosin Pigment

The prodigiosin pigment was produced by *Serratia plymuthica*, gently provided by Peter Askew (Industrial Microbiological Services Ltd., Hampshire, UK), using a Peptone Glycerol Phosphate (PGP) medium (5 g/L peptone, 10 mL/L glycerol, 2 g/L K_2_HPO_4_, and 15 g/L agar-agar) at 20 °C in the absence of light [12]. The pigment was directly collected from the medium-agar plates and used as a crude gel prodigiosin pigment.

### 4.3. Preparation of the Crude Gel Prodigiosin Pigment Solution and Dyeing Process

The crude gel prodigiosin pigment bath solution was prepared in an acidified ethanol/distilled water (1:99) system with a concentration of 30% (*w/v*). In turn, the dyeing processes were performed using a 4 cm × 10 cm multifiber fabric composed of acetate, cotton, nylon, polyester, acrylic, and wool on the Datacolor AHIBA IR equipment (Datacolor company, Lawrenceville, NJ, USA) using the exhaustion method. The dyeing duration (60 min), bath ratio (1:50), rate of temperature rise (2 °C/min), and stirring rate (20 rpm) were kept constant throughout the experiments. After the dyeing processes, the multifiber fabrics were rinsed with distilled water twice and then dried at room temperature.

#### Optimization of Dyeing Conditions

Step 1: The effect of temperature

In order to evaluate the influence of temperature in the dyeing process, multifiber fabrics were dyed with the crude gel prodigiosin pigment at temperatures of 40 and 60 °C.

Step 2: The effect of the dyeing aids’ addition

− The addition of salts: salts that are often used for dyeing textile fibers, like NaOH (12 mL/L) and Na_2_S_2_O_4_ (4 g/L), were added to the aqueous crude gel prodigiosin pigment bath in order to evaluate the effectiveness of these salts in the dyeing process.
− The addition of mordants: The mordant process was performed based on the simultaneous mordanting methodology using two different mordants: FeSO_4_, a metallic mordant, and L-Cys, an amino acid bio-mordant, at 1.0%, 3.0%, and 5.0% *owf* under the same dyeing conditions. Moreover, the effect of the crude gel prodigiosin pigment bath’s pH on dyeing the multifiber fabrics was evaluated at pH = 4.0 and pH = 8.3 in order to achieve an acidic pH during dyeing with the FeSO_4_ metallic mordant and an alkaline pKa of the bio-mordant L-Cys, respectively.

Step 3: The effect of the use of gel-based deep eutectic solvents (DESs) as a prodigiosin dyeing medium

Firstly, the ChCl (hydrogen bond donor—HBD) was combined with LA (hydrogen bond acceptors—HBA) in a 1:2 molar ratio in order to produce the gel-based DES mixture. The DES components were added to a sealed lab flash and heated at 80 °C under magnetic stirring until a perfectly clear and transparent gel–liquid was formed with a pH = 0.9. Then, 30% (*w/v*) of the crude gel prodigiosin pigment, dissolved in the pre-prepared gel-based ChCl/LA (1:2) DES with a pH = 1.7, was used as a green dyeing medium. The multifiber fabric was dyed under the same dyeing conditions, namely at a liquor ratio of 1:50, with a dyeing temperature and time of 60 °C and 60 min, respectively.

### 4.4. Characterization of the Dyed Fabrics

#### 4.4.1. Color Strength Measurements of Dyed Fabrics

The color yield of the crude gel prodigiosin pigment-dyed multifiber fabrics was assessed based on the *K/S* from the reflectance values using a Datacolor 110 spectrophotometer (Datacolor company, Lawrenceville, NJ, USA) at 535 nm. The *K/S* values were determined using the Kubelka–Munk Equation (1):

(1) $$K/S=\frac{{\left(1-R\right)}^{2}}{2R}$$

where *R* represents the observed reflectance of the dyed fabrics, *K* is the absorption coefficient, and *S* is the scattering coefficient. The *K/S* values were reported as the average of at least three measurements at different positions (*n* = 3). The different measurements at various points of the samples were also used to verify the *dE*.

#### 4.4.2. Analysis of the Surface Morphology of Dyed Fabrics through SEM

The surface morphology of the undyed and dyed fabrics with superior performance, namely the wool and nylon fabrics dyed with 3.0% *owf* of L-Cys at pH = 8.3, and the nylon dyed using the gel-based ChCl/LA (1:2) DES as a dyeing medium, was characterized through scanning electron microscopy (SEM) using a Hitachi S-3400N Scanning Electron Microscope (Hitachi, Tokyo, Japan) at an acceleration voltage of 20 kV. The fabrics were sputtered with gold using a Quorum Q150R ES sputter coater (Quorum Technologies Ltd., Laughton, East Sussex, UK) before the morphology analysis.

#### 4.4.3. Evaluation of Fastness Properties of Dyed Fabrics

Upon finishing the dyeing processes, the fastness to washing and light was studied. For the color fastness to washing, the dyed fabrics with superior performance, namely the wool and nylon fabrics dyed with 3.0% *owf* of L-Cys at pH = 8.3, and the nylon dyed using the gel-based ChCl/LA (1:2) DES as a dyeing medium, were washed with a commercial ECE detergent (4 g/L) according to ISO 105-C06:2010 for 30 min at 40 °C, using a sample dyeing machine Linitest Device. After washing, the samples were rinsed in distilled water and dried at room temperature.

The *∆E* of the samples before and after washing was determined based on the colorimetric properties using the CIELab values (L*, a*, and b*) from Equation (2):

(2) $$∆E={\left({∆a*}^{2}+{∆b*}^{2}+{∆L*}^{2}\right)}^{1/2}$$

where a* represents redness and greenness, b* indicates yellowness and blueness, and L* shows lightness from black (0) to white (100).

The evaluation of the *∆E* was carried out in the Datacolor spectraflash SF300 spectrophotometer, and the fastness index (*∆Color*) in the Color-Chex lightbox, using the CIELab gray-scale and the CIE illuminant D65.

In turn, for the light fastness, the nylon fabric dyed with the crude gel prodigiosin pigment with the addition of the 3.0% *owf* of the L-Cys bio-mordant at pH = 8.3 and using the gel-based ChCl/LA (1:2) DES as a dyeing medium was tested following a method adapted from ISO 105-B02:2014. Briefly, the nylon samples (7.5 cm × 7.5 cm) were exposed to artificial daylight and blacklight in a Color-Chex lightbox for 0, 16, and 24 h. After that, the fading degree was assessed by measuring the sample *K/S* using a Datacolor 110 spectrophotometer (Datacolor company, USA) and the *%D* was determined using Equation (3):

(3) $$\%D=\frac{{K/S}_{i}-{K/S}_{f}}{{K/S}_{i}}\times 100$$

where *K/S_i_* is the sample color strength before exposure to artificial lights and *K/S_f_* is the sample color strength after exposure to artificial daylight or blacklight. 

#### 4.4.4. Evaluation of the Antibacterial Activity of Dyed Fabrics

The antibacterial efficiency of the nylon dyed with the crude gel prodigiosin pigment, with and without the addition of 3.0% *owf* L-Cys bio-mordant at pH = 8.3, and using the gel-based ChCl/LA (1:2) DES as a dyeing medium, was evaluated against *Staphylococcus aureus* ATTC 6538 (*S. aureus*) and *Pseudomonas aeruginosa* PA25 (*P. aeruginosa*) according to Japanese Industrial Standard JIS L 1902:2002, a standard method that is widely used to test the antibacterial activity of textiles. Briefly, a bacterial suspension (1 ± 0.3 × 10^5^ CFU/mL) was prepared and inoculated on nylon square samples. Then, the samples were subjected to vigorous vortex mixing for 30 s in a neutralizing solution and serial dilutions were prepared with 0.85 (*w/v*) NaCl, plated in agar plates, and incubated for 18–24 h at 37 °C. The antimicrobial activity was quantitatively expressed as the percentage of bacterial growth inhibition (*% Inhibition*) using Equation (4):

(4) $$\%\;Inhibition=\frac{C\;-\;A}{C}\times 100$$

where *C* is the average value of the CFU of the undyed nylon sample, and *A* is the average value of the CFU of the nylon dyed with the crude gel prodigiosin pigment with and without the addition of 3.0% *owf* L-Cys at pH = 8.3 and using the gel-based ChCl/LA (1:2) DES dyeing medium.

## Figures and Tables

**Table 1:** Color Strength (*K/S*) measurements and the dyeing equalization (*dE*) values on multifiber dyeing with the crude gel prodigiosin pigment at 40 °C and 60 °C (a) and after the addition of salts (b).

(a)			**Temperature (°C)**		(b)		**Salts**	
			**40**	**60**				
	Acetate	*K/S*	0.49	0.47		Acetate	*K/S*	0.59
		*dE*	0.10	0.07			*dE*	0.19
	Cotton	*K/S*	0.78	0.70		Cotton	*K/S*	0.25
		*dE*	0.10	0.08			*dE*	0.16
	Nylon	*K/S*	1.17	1.20		Nylon	*K/S*	1.25
		*dE*	0.16	0.04			*dE*	0.14
	Polyester	*K/S*	0.68	0.72		Polyester	*K/S*	0.39
		*dE*	0.09	0.05			*dE*	0.07
	Acrylic	*K/S*	0.51	0.30		Acrylic	*K/S*	0.14
		*dE*	0.06	0.03			*dE*	0.10
	Wool	*K/S*	1.56	1.38		Wool	*K/S*	1.60
		*dE*	0.12	0.05			*dE*	0.05

**Table 2:** *K/S* measurements and *dE* values on multifiber dyeing with the crude gel prodigiosin pigment after the addition of mordants (FeSO_4_ and L-Cys) at 1.0, 3.0, and 5.0% *owf*.

		Mordants					
		Ferrous Sulphate (FeSO_4_)			L-Cysteine (L-Cys)		
		1.0%	3.0%	5.0%	1.0%	3.0%	5.0%
Acetate	*K/S*	0.75	1.65	6.43	0.47	0.63	0.70
	*dE*	0.15	0.08	0.17	0.13	0.08	0.10
Cotton	*K/S*	0.55	0.90	1.23	0.59	0.86	0.94
	*dE*	0.14	0.11	0.43	0.04	0.04	0.09
Nylon	*K/S*	1.39	2.02	6.81	1.12	1.72	1.61
	*dE*	0.09	0.09	0.16	0.06	0.02	0.07
Polyester	*K/S*	0.48	0.60	0.77	0.68	0.76	0.91
	*dE*	0.10	0.15	0.30	0.07	0.04	0.19
Acrylic	*K/S*	0.27	0.66	0.92	0.19	0.21	0.35
	*dE*	0.09	0.06	0.55	0.05	0.02	0.12
Wool	*K/S*	1.47	2.44	2.06	1.69	2.08	2.48
	*dE*	0.10	0.11	0.96	0.06	0.04	0.11

**Table 3:** *K/S* measurements and *dE* values on multifiber dyeing with the crude gel prodigiosin pigment at pH = 4.0 and pH = 8.3 with and without adding mordants (3.0% *owf* FeSO_4_ and 3.0% *owf* L-Cys).

		pH					
		4.0			8.3		
		Control	3.0% *owf* FeSO_4_	3.0% *owf* L-Cys	Control	3.0% *owf* FeSO_4_	3.0% *owf* L-Cys
Acetate	*K/S*	1.71	3.81	1.53	0.51	1.01	0.58
	*dE*	1.21	0.19	1.16	0.11	0.12	0.11
Cotton	*K/S*	0.86	1.46	0.83	0.90	1.21	0.97
	*dE*	0.83	0.62	0.80	0.17	0.23	0.13
Nylon	*K/S*	1.73	3.30	1.59	1.87	3.67	2.30
	*dE*	1.01	0.13	1.00	0.09	0.11	0.05
Polyester	*K/S*	1.10	1.11	0.97	0.95	1.28	0.97
	*dE*	0.41	0.22	0.33	0.12	0.26	0.11
Acrylic	*K/S*	0.57	1.04	0.61	0.24	0.42	0.26
	*dE*	0.17	0.23	0.15	0.17	0.20	0.11
Wool	*K/S*	2.33	3.22	2.04	2.38	3.11	2.49
	*dE*	0.70	0.35	0.84	0.25	0.33	0.27

**Table 4:** *K/S* measurements and *dE* values for multifiber fabric using the gel-based DES as a prodigiosin dyeing medium.

Gel-Based Deep Eutectic Solvent (DES)		
ChCl/LA (1:2)		
Acetate	*K/S*	2.76
	*dE*	0.02
Cotton	*K/S*	1.60
	*dE*	0.02
Nylon	*K/S*	3.33
	*dE*	0.01
Polyester	*K/S*	1.22
	*dE*	0.01
Acrylic	*K/S*	1.55
	*dE*	0.01
Wool	*K/S*	1.60
	*dE*	0.01

**Table 5:** The washing fastness properties of the nylon and wool with 3.0% *owf* L-Cys at pH = 8.3 and the nylon dyed using the gel-based ChCl/LA (1:2) DES.

	∆L*	∆a*	∆b*	∆E*	Fastness Index
					*∆Color*
Prodigionsin_3.0% *owf* L-Cys_Nylon_pH = 8.3	1.66	−0.77	2.64	3.21	3
Prodigiosin_3.0% *owf* L-Cys_Wool_pH = 8.3	3.31	−6.56	2.86	7.88	2
Prodigiosin_gel-based ChCl/LA (1:2) DES_Nylon	11.12	−1.15	1.09	10.77	1–2

## Data Availability

The data that support the findings of this study are included in the article.

## Abbreviations

***%D***, Discoloration percentage; ***∆Color***, color fastness; ***∆E****, color difference; **BHI**, brain heart infusion agar; **ChCl**, choline chloride; ***dE***, dyeing equalization; **DES**, deep eutectic solvent; **DESs**, deep eutectic solvents; **DMSO**, dimethyl sulfoxide; **FeSO_4_**, ferrous sulphate; **HBA**, hydrogen bond acceptors; **HBD**, hydrogen bond donor; **HCl**, hydrochloric acid; **K_2_HPO_4_**, dipotassium hydrogen phosphate; ***K/S***, color Strength; ***L***, lightness; **LA**, lactic acid; ***L-Cys***, L-Cysteine; **NA**, nutrient agar; **NaOH**, sodium hydroxide; **Na_2_S_2_O_4_**, sodium hydrosulphite; **NB**, nutrient broth; ***owf***, over the weight of the fiber; ***P. aeruginosa***, *Pseudomonas aeruginosa*; **PGP**, peptone glycerol phosphate; **PVA**, polyvinyl alcohol; ***S. aureus***, *Staphylococcus aureus*; **SEM**, scanning electron microscopy; **SH**, thiol groups.

## References

[1] Haji A., Naebe M. (2020). Cleaner Dyeing of Textiles Using Plasma Treatment and Natural Dyes: A Review. J. Clean. Prod..

[2] Kramar A., Kostic M.M. (2022). Bacterial Secondary Metabolites as Biopigments for Textile Dyeing. Textiles.

[3] Metwally R.A., El Sikaily A., El-Sersy N.A., Ghozlan H.A., Sabry S.A. (2021). Antimicrobial Activity of Textile Fabrics Dyed with Prodigiosin Pigment Extracted from Marine *Serratia rubidaea* RAM_Alex Bacteria. Egypt. J. Aquat. Res..

[4] Silva P.M.d.S., Fiaschitello T.R., de Queiroz R.S., Freeman H.S., da Costa S.A., Leo P., Montemor A.F., da Costa S.M. (2020). Natural Dye from *Croton urucurana* Baill. Bark: Extraction, Physicochemical Characterization, Textile Dyeing and Color Fastness Properties. Dyes Pigm..

[5] Amutha K., Grace Annapoorani S., Sudhapriya N. (2020). Dyeing of Textiles with Natural Dyes Extracted from *Terminalia arjuna* and *Thespesia populnea* Fruits. Ind. Crops Prod..

[6] Zheng M., Sun Y., Li C., Lu Y., Dai Y., Wang Z. (2023). A Novel and Eco-Friendly Approach for Dyeing Polyester Fabrics by Liquid Disperse Dyes Treated with Deep Eutectic Solvent. Color. Technol..

[7] Jiang Z., Cui Y., Zheng G., Wei Y., Wang Q., Zhou M., Wang P., Yu Y. (2022). An Innovative, Low-Cost and Environment-Friendly Approach by Using a Deep Eutectic Solvent as the Water Substitute to Minimize Waste in the Textile Industry and for Better Clothing Performance. Green Chem..

[8] Aruldass C.A., Aziz A., Venil C.K., Khasim A.R., Ahmad W.A. (2016). Utilization of Agro-Industrial Waste for the Production of Yellowish-Orange Pigment from *Chryseobacterium artocarpi* CECT 8497. Int. Biodeterior. Biodegrad..

[9] Panesar R., Kaur S., Panesar P.S. (2015). Production of Microbial Pigments Utilizing Agro-Industrial Waste: A Review. Curr. Opin. Food Sci..

[10] Venil C.K., Devi P.R., Ahmad W.A. (2020). Agro-Industrial Waste as Substrates for the Production of Bacterial Pigment. Valorisation of Agro-Industrial Residues—Volume I: Biological Approaches.

[11] Amorim L.F.A., Fangueiro R., Gouveia I.C. (2022). Characterization of Bioactive Colored Materials Produced from Bacterial Cellulose and Bacterial Pigments. Materials.

[12] Amorim L.F.A., Mouro C., Riool M., Gouveia I.C. (2022). Antimicrobial Food Packaging Based on Prodigiosin-Incorporated Double-Layered Bacterial Cellulose and Chitosan Composites. Polymers.

[13] Arivizhivendhan K.V., Mahesh M., Boopathy R., Swarnalatha S., Regina Mary R., Sekaran G. (2018). Antioxidant and Antimicrobial Activity of Bioactive Prodigiosin Produces from *Serratia marcescens* Using Agricultural Waste as a Substrate. J. Food Sci. Technol..

[14] Bhagwat A., Padalia U. (2020). Optimization of Prodigiosin Biosynthesis by *Serratia marcescens* Using Unconventional Bioresources. J. Genet. Eng. Biotechnol..

[15] Elkenawy N.M., Yassin A.S., Elhifnawy H.N., Amin M.A. (2017). Optimization of Prodigiosin Production by *Serratia marcescens* Using Crude Glycerol and Enhancing Production Using Gamma Radiation. Biotechnol. Rep..

[16] Lazic J., Skaro Bogojevic S., Vojnovic S., Aleksic I., Milivojevic D., Kretzschmar M., Gulder T., Petkovic M., Nikodinovic-Runic J. (2022). Synthesis, Anticancer Potential and Comprehensive Toxicity Studies of Novel Brominated Derivatives of Bacterial Biopigment Prodigiosin from *Serratia marcescens* ATCC 27117. Molecules.

[17] Nguyen S.L.T., Nguyen T.C., Do T.T., Vu T.L., Nguyen T.T., Do T.T., Nguyen T.H.T., Le T.H., Trinh D.K., Nguyen T.A.T. (2022). Study on the Anticancer Activity of Prodigiosin from Variants of *Serratia marcescens* QBN VTCC 910026. Biomed. Res. Int..

[18] Picha P., Kale D., Dave I., Pardeshi S. (2015). Comparative Studies on Prodigiosin Production by *Serratia marcescens* Using Various Crude Fatty Acid Sources-Its Characterization and Applications. Int. J. Curr. Microbiol. Appl. Sci..

[19] Ren Y., Gong J., Fu R., Li Z., Li Q., Zhang J., Yu Z., Cheng X. (2017). Dyeing and Antibacterial Properties of Cotton Dyed with Prodigiosins Nanomicelles Produced by Microbial Fermentation. Dyes Pigm..

[20] Venil C.K., Dufossé L., Velmurugan P., Malathi M., Lakshmanaperumalsamy P. (2021). Extraction and Application of Pigment from *Serratia marcescens* SB08, an Insect Enteric Gut Bacterium, for Textile Dyeing. Textiles.

[21] Zhao Y., Cheng Q., Shen Z., Fan B., Xu Y., Cao Y., Peng F., Zhao J., Xue B. (2020). Structure of Prodigiosin from *Serratia marcescens* NJZT-1 and Its Cytotoxicity on TSC2-Null Cells. Food Sci. Technol..

[22] Nguyen T.H., Wang S.L., Nguyen V.B. (2022). Recent Advances in Eco-Friendly and Scaling-Up Bioproduction of Prodigiosin and Its Potential Applications in Agriculture. Agronomy.

[23] Liu X., Shen J., Wang Y., Wu J., Lu Z. (2011). Biological Dye Containing Prodigiosins, Preparation Method Thereof and Application Thereof.

[24] Alihosseini F., Ju K.S., Lango J., Hammock B.D., Sun G. (2008). Antibacterial Colorants: Characterization of Prodiginines and Their Applications on Textile Materials. Biotechnol. Prog..

[25] Kramar A., Ilic-Tomic T., Petkovic M., Radulović N., Kostic M., Jocic D., Nikodinovic-Runic J. (2014). Crude Bacterial Extracts of Two New *Streptomyces* sp. Isolates as Bio-Colorants for Textile Dyeing. World J. Microbiol. Biotechnol..

[26] Liu X., Wang Y., Sun S., Zhu C., Xu W., Park Y., Zhou H. (2013). Mutant Breeding of *Serratia marcescens* Strain for Enhancing Prodigiosin Production and Application to Textiles. Prep. Biochem. Biotechnol..

[27] Vaidyanathan J., Bhathena-Langdana Z., Adivarekar R.V., Nerurkar M. (2012). Production, Partial Characterization, and Use of a Red Biochrome Produced by *Serratia sakuensis* subsp. Nov Strain KRED for Dyeing Natural Fibers. Appl. Biochem. Biotechnol..

[28] Namazkar S., Garg R., Ahmad W.Z., Nordin N. (2013). Production and Characterization of Crude and Encapsulated Prodigiosin Pigment. Int. J. Chem. Sci. Appl..

[29] Burkinshaw S.M., Kumar N. (2009). The Mordant Dyeing of Wool Using Tannic Acid and FeSO_4_, Part 1: Initial Findings. Dyes Pigm..

[30] Mongkholrattanasit R., Kryštůfek J., Wiener J., Viková M. (2011). UV Protection Properties of Silk Fabric Dyed with Eucalyptus Leaf Extract. J. Text. Inst..

[31] Uddin M.G. (2014). Effects of Different Mordants on Silk Fabric Dyed with Onion Outer Skin Extracts. J. Text..

[32] Cai C., Li F., Liu L., Tan Z. (2019). Deep Eutectic Solvents Used as the Green Media for the Efficient Extraction of Caffeine from Chinese Dark Tea. Sep. Purif. Technol..

[33] Torres-Vega J., Gómez-Alonso S., Pastene-Navarrete E., Pérez-Navarro J. (2020). Green Extraction of Alkaloids and Polyphenols from *Peumus boldus* Leaves with Natural Deep Eutectic Solvents and Profiling by HPLC-PDA-IT-MS/MS and HPLC-QTOF-MS/MS. Plants.

[34] Shawky E., Takla S.S., Hammoda H.M., Darwish F.A. (2018). Evaluation of the Influence of Green Extraction Solvents on the Cytotoxic Activities of *Crinum* (Amaryllidaeae) Alkaloid Extracts Using in-Vitro-in-Silico Approach. J. Ethnopharmacol..

[35] Kerwin B.A., Remmele R.L. (2007). Protect from Light: Photodegradation and Protein Biologics. J. Pharm. Sci..

[36] Koch R., Fränz J. (1960). Der Einfluss Des Lichtes Und Ionisierender Strahlung Auf Die Autoxydation von Cystein Und Die Reaktion Zwischen Cystein Und 3, 4-Benzpyren. Int. J. Radiat. Biol..

[37] Ren Y., Gong J., Fu R., Li Z., Yu Z., Lou J., Wang F., Zhang J. (2017). Dyeing and Functional Properties of Polyester Fabric Dyed with Prodigiosins Nanomicelles Produced by Microbial Fermentation. J. Clean. Prod..

[38] Gouveia I.C. (2010). Nanobiotechnology: A New Strategy to Develop Non-Toxic Antimicrobial Textiles for Healthcare Applications. J. Biotechnol..

[39] Mouro C., Martins R., Gomes A.P., Gouveia I.C. (2023). Upcycling Wool Waste into Keratin Gel-Based Nanofibers Using Deep Eutectic Solvents. Gels.

[40] Akbar N., Khan N.A., Ibrahim T., Khamis M., Khan A.S., Alharbi A.M., Alfahemi H., Siddiqui R. (2023). Antimicrobial Activity of Novel Deep Eutectic Solvents. Sci. Pharm..

[41] Giteru S.G., Ramsey D.H., Hou Y., Cong L., Mohan A., Bekhit A.E.D.A. (2023). Wool Keratin as a Novel Alternative Protein: A Comprehensive Review of Extraction, Purification, Nutrition, Safety, and Food Applications. Compr. Rev. Food Sci. Food Saf..

